# Sirtuin 1 and endothelial glycocalyx

**DOI:** 10.1007/s00424-020-02407-z

**Published:** 2020-06-03

**Authors:** Mark Lipphardt, Jong Wook Song, Michael S Goligorsky

**Affiliations:** 1 Renal Research Institute, New York Medical College at the Touro University, Valhalla, NY USA; 2 Department of Nephrology and Rheumatology, Göttingen University Medical Center, Georg August University, Robert-Koch-Straße 40, 37075 Göttingen, Germany; 3 Department of Anesthesiology and Pain Medicine, Yonsei University College of Medicine, Seoul, South Korea

**Keywords:** Glycocalyx, Sirtuin 1, Syndecan-4, Sheddases, NF-kB, Oxidative stress

## Abstract

Sirtuin1 deficiency or reduced activity comprises one of the hallmarks of diseases as diverse as chronic cardiovascular, renal, and metabolic, some malignancies, and infections, as well as aging-associated diseases. In a mouse model of endothelium-limited defect in sirtuin 1 deacetylase activity, we found a dramatic reduction in the volume of endothelial glycocalyx. This was associated with the surge in the levels of one of key scaffolding heparan sulfate proteoglycans of endothelial glycocalyx, syndecan-4, and specifically, its extracellular domain (ectodomain). We found that the defect in endothelial sirtuin 1 deacetylase activity is associated with (a) elevated basal and stimulated levels of superoxide generation (via the FoxO1 over-acetylation mechanism) and (b) increased nuclear translocation of NF-kB (via p65 over-acetylation mechanism). These findings laid the foundation for the proposed novel function of sirtuin 1, namely, the maintenance of endothelial glycocalyx, particularly manifest in conditions associated with sirtuin 1 depletion. In the forthcoming review, we summarize the emerging conceptual framework of the enhanced glycocalyx degradation in the states of defective endothelial sirtuin 1 function, thus explaining a broad footprint of the syndrome of endothelial dysfunction, from impaired flow-induced nitric oxide production, deterrent leukocytes infiltration, increased endothelial permeability, coagulation, and pro-inflammatory changes to development of microvascular rarefaction and progression of an underlying disease.

## Introduction

Sirtuin family (SIRT) of protein and histone deacetylases is represented by seven mammalian SIRT proteins (SIRT1-SIRT7). Sirtuins have been the subject of multitude of investigations which have demonstrated that SIRT proteins are responsible for diverse cell functions and are capable of protection against several diseases like diabetes, cancer, or cardiovascular diseases [50, 53, 63]. There are approximately 500 different known target proteins for SIRT-deacetylation. SIRT1, the founding member of the family [39], is an NAD-dependent deacetylase participating in chromatin regulation (by deacetylating H3K9, H3K56, H4K16, H1K26, SUV39H1, p300, and PCAF); DNA repair (HDAC1, PARP1, p53, KU70, NBS1, E2F1, RB, XPA, WRN, survivin, β-catenin, MYC, NF-κB, and TOPBP1), and cell metabolism (PGC1α, FOXO1, FOXO3A, FOXA2, CRCT1, CRCT2, PPARα, PPARγ, LXR, FXR, RARβ, SREBP1C, SREBP2, HNF4α, HIF1α, HIF2α, CREB, NKX2–1, STAT3, TFAM, MYOD, NHLH2, UCP2, TSC2, eNOS, LKB1, SMAD7, AKT, ATG5, ATG7, ATG8, 14-3-3ζ, PGAM1, ACECS1, PTP1B, and S6K1) [17]. Studies of the role of SIRT1 in endothelial cells were significantly accelerated by the creation of SIRT1^endo^-/- mice [97] which demonstrated that this deacetylase participates in angiogenesis.

We have recreated and further explored this mouse model [22, 76, 123] to demonstrate development of premature senescence of endothelial cells, rarefaction of renal peritubular microvascular network, diastolic dysfunction, reduced expression of the membrane-tethered matrix metalloproteinase MMP-14, defective acetylcholine-induced vasorelaxation, and propensity toward tubulointerstitial fibrosis—all hallmarks of developing endothelial cell dysfunction. The fact that dysfunctional endothelial cells can trigger fibrosis alluded to the possibility of secretory products of such cells inducing fibroblast activation and prompted us to perform an unbiased proteomic screen of the secretome of SIRT1-deficient vis-à-vis control endothelial cells isolated from renal microvasculature [70–72]. Among various differentially expressed proteins in the aberrant secretome, syndecan-4 ectodomain was prominently present [73]. Syndecan-4 is a major proteoglycan of endothelial cell glycocalyx. Hence, in parallel studies of SIRT1^endo^-/- mice, Song et al. [110] demonstrated by dual-fluorophore dilution technique that endothelial glycocalyx of these mice is disintegrated. Based on these findings, we deduced that SIRT1 may have another, yet unidentified, function in maintaining endothelial glycocalyx integrity. This review summarizes our and others’ most recent findings on this subject. It should be noted that this review is not intended to illustrate the entire spectrum of glycocalyx components and functions, rather it is limited to exhibit our hypothesis that SIRT1-induced deacetylation is crucial for the maintenance of endothelial glycocalyx.

## Endothelial glycocalyx

The endothelial glycocalyx (EG) can be defined as a layer with a high amount of carbohydrates which covers the vascular endothelium. It is coated with a carbohydrate-rich layer of an average thickness of 0.2–2 um and consisting of hyaluronic acid (HA) cords reaching 1 um in length, heparan sulfate (HS) chains reaching in length 200 nm, and comprising 50–90% of endothelial glycosaminoglycans, with an admixture of dermatan, keratan, and chondroitin sulfates [103]. The high degree of sulfation of these components provides EG with a net negative charge [103]. Among those mentioned glycosaminoglycans, the most common ones are HS followed by chondroitin sulfate and HA, although the levels of each glycosaminoglycan depend highly on various current stimuli [99]. HA differs from the other glycosaminoglycans, since it has no linkage to a core protein and it binds to the osteopontin receptor CD44 [87]. With the newest technique, the use of super-resolution optical microscopy (STORM), the study group of Fan et al. was able to visualize HA as long molecules forming a hexagonal network which covers the endothelial lumen. HS, on the other hand, was visualized as a shorter molecule with a straight positioning to the cell surface [37]. The HA network plays a major role in the stability and function of the EG, as passage through the EG is regulated by HA [126]. Moreover, endothelial mechanosensing and the preservation of endothelial quiescence are in need of the presence of HA [98]. HS acts mainly as a mechanosensor arbitrating the regular release of NO as shown by Florian et al. [38].

The membrane-tethered scaffold for these glycosaminoglycans consists of two families of proteoglycans: syndecans 1–4 (single membrane-spanning domain) and glypicans 1–6 (glycosylphosphatidylinositol-anchored) [103]. Since there are multiple ways of modifications of the glycosaminoglycan chains, the diversity of the glycosaminoglycans results in the alternation of specific protein binding, in the alternation of protein functions, and in the modulation of vascular permeability [103]. Consequently, the EG creates a dynamic balance between itself and the luminal components, alternating its composition and thickness [103].

In addition, several glycoprotein families (selectins, integrins, and immunoglobulins) are present in EG. Their main role of action is the regulation of cell recruitment from the bloodstream. Selectins present in EG are E-selectin and P-selectin with their main action in the field of cell interaction between leukocytes and the endothelium [111]. Especially E-selectin is ovexpressed in endothelial cells after stimulation by cytokines [57]. Integrins can be described as molecules composed of non-covalently bound α and β subunits [103]. Luminally endothelial cells express integrin αVβ3, mediating the cell interaction between the endothelial cell and platelets [9]. The other integrins expressed at EG are associated with binding to the basement membrane and interact with laminin, fibronectin, and collagen [103]. The immunoglobulins are divided in a cytoplasmic, a transmembrane, and an extracellular part, acting as ligands for integrins and mediate leukocyte homing to the endothelium [103].

EG provides a repository for diverse biologically active molecules, as it incorporates and interacts with extracellular superoxide dismutase (SOD), xanthine oxidoreductase, interleukins, like IL2, IL5, IL7, IL8, and IL12, low density lipoprotein (LDL) and LDL lipase, bFGF, VEGF, and TGF-beta, and several regulators of coagulation, like antithrombin III, heparin cofactor II, and tissue pathway factor inhibitor [103]. EG is a guardian of endothelial cell homeostatic functions. Due to its unique location, this structure provides a passive barrier to water and solutes (regulation of vascular permeability), and to the interaction between circulating cells and the endothelial cells (regulation of leukocyte trafficking) [5]. It also serves as a sensor of mechanical forces, such as shear stress and pressure, and shields cell surface receptors preventing their hyper-activation [5, 40].

This structure is, however, quite vulnerable and tends to disintegrate under the influence of various stressors, such as endotoxins, ischemia/hypoxia/reperfusion, oxidative stress, among others [107]. It also leads to hyper-activation of plasma membrane receptors left exposed to respective unhindered ligands, with further activation of endothelial cells and propagation of danger signaling [27]*.* The degradation of EG is also accompanied by the compromised anti-coagulant properties of this layer, increased endothelial permeability, reduced antioxidant barrier, enhanced transmigration of pro-inflammatory cells, impaired mechanotransduction, and endothelial nitric oxide synthase activity [2, 27, 107]. In acute kidney injury induced by ischemia/reperfusion, sepsis, and/or kidney transplantation, EG is impaired both in experimental animals and in humans [19, 47, 84, 95, 108, 109, 134].

Another condition frequently associated with the degradation of EG is diabetic nephropathy. Deckert and colleagues [25] were the first to show that the de novo synthesis of heparan sulfate was reduced in fibroblasts isolated from diabetes patients with albuminuria, but not from those without albuminuria or control healthy subjects, and formulated a hypothesis that the loss of EG is a prerequisite for the developing diabetic nephropathy. Recently, upregulation of endothelin-1 in diabetes was incriminated in the induction of heparanase in podocytes, resulting in impairment of glomerular EG [43]. This is in agreement with studies by different investigators who have demonstrated the loss of glycocalyx integrity in diabetes mellitus [85, 90, 91]. Considering the role of EG in endothelial cell function and dysfunction [133], its putative dependence on SIRT1 expression and activity, both impaired in the above pathologic conditions, gains additional import. Our recent unbiased proteomic studies of microvascular endothelial cells expressing deacetylation-deficient SIRT1 have revealed upregulation of syndecan-4, and, specifically, its ectodomain. Scenarios tentatively explaining this finding are briefly summarized below.

## NF-κB as a target for SIRT1 deacetylation

It has been well-documented that SIRT1 is a negative regulator of inflammation, in part due to its effects on nuclear factor kappa-light-chain-enhancer of activated B cells (NF-kB), one of the target proteins for SIRT1-deacetylation [130]. In mammals, the following members of the NF-κB family have been described: NF-κB1 (p105/p50), NF-κB2 (p100/p52), RelA (p65), RelB, and c-Rel [10, 45]. NF-κB is known for its regulatory effects on transcription of DNA, cytokine production, and cell survival [13]. NF-κB usually forms dimers, which is necessary for binding DNA. One typical structure of NF-κB is the p50-p65 dimer (NF-κB1/RelA) [20].

In order to unfold its transcriptional activity, NF-κB needs to translocate into the nucleus. In an inactive state NF-κB remains in the cytoplasm and is bound to specific inhibitors, the Iκ-B proteins (IκBa, IκBb and IκBg), which, in turn, bind to the Rel homology domain (RHD) of NF-κB and therefore interfere with its nuclear translocation [10, 121]. Hence the activation of NF-κB is linked to the release of its inhibtors. Pro-inflammatory cytokines induce the activation of the IκB kinase complex, releasing NF-κB from its inhibitors and consequently leading to NF-κB nuclear translocation [10, 121].

**Fig. 1:**
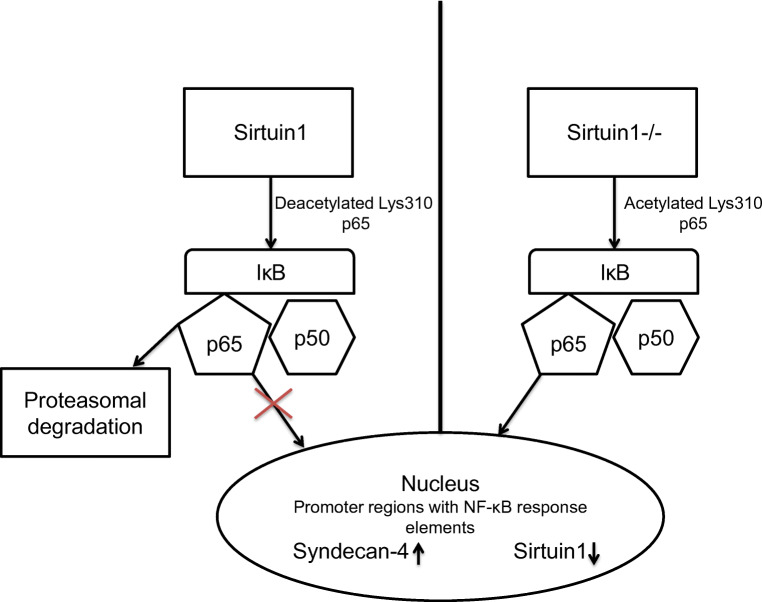
Interaction between Sirtuin1 and NF-κB, Sirtuin1 deacetylates p65 at Lys310 disabling the transcriptional activity of p65 and results in the proteosomal degradation of p65. If SIRT1 is inhibited or deficient, p65 remains in its acetylated form, and therefore p65 is able to release itself from IκB and translocates to the nucleus. In the nucleus p65 induces the transcription of syndecan-4 and reduces the transcription of Sirtuin1

NF-κB is a target protein for SIRT-deacetylation (Fig. 1). In fact, SIRT1 binds to p65 protein disabling its transcriptional activity by deacetylating p65 at Lys^310^ [12, 131]. Consistent with this, induction of SIRT1 results in the inhibition of NF-κB-dependent inflammatory pathway [93, 131] and vice versa, which reduced activation of SIRT1 that leads to enhanced NF-κB signaling [58].

This relation between SIRT1 and NF-κB becomes a main concern when SIRT1 is inhibited exclusively in the endothelium, which has been demonstrated to represent an excellent model of global endothelial dysfunction [123]. In case of SIRT1 inhibition in endothelial cells, the increased NF-κB signaling could lead to the enhanced degradation of the EG in part due to induced shedding of EG proteoglycans and activation of heparanases.

## Syndecan-4: a proteoglycan of the endothelial glycocalyx

Proteoglycans represent one of the essential components of the EG, which is also true for the underlying basement membrane of endothelial cells [125]. Among those proteoglycans there is the family of syndecans. Syndecans are transmembrane receptors with autonomous and combined signal capacity which have mostly heparan sulfate glycosaminoglycans covalently bound to their extracellular domain [23, 24, 36]. The family of syndecans includes four members: syndecans1–4. Syndecan-1 is typically found in epithelial, endothelial, and plasma cells, whereas syndecan-2 is rich in endothelial and mesenchymal cells and syndecan-3 is mostly found in neural crest-derived cells [64, 118]. Syndecan-4, however, is the only family member with a ubiquitous distribution and is highly expressed in endothelial and epithelial cells in the kidney and other organs [125]. In cultured endothelial cells, inflammatory mediators such as lipopolysaccharide (LPS) and interleukin 1β (IL-1β) syndecan-4 expression showed a rapid increase, while Syndecan-1 and -2 expression decreased and syndecan-3 was unaffected [125] implying that this proteoglycan may represent a key proinflammatory sensor of endothelial cells. Syndecan-4 is a target of TNF-alpha-induced matrix metalloproteinase-9 leading to degradation of glycocalyx [101]. Syndecan-4 has been found to counteract effects of eNOS and serve as an enhancer of angiopoitin-2 secretion leading to antiangiogenesis [56]. The spectrum of Syndecan-4 signaling properties has been comprehensively reviewed [34]:

One of the main functions of syndecan-4 is to serve as a co-receptor for heparin-binding growth factors, such as fibroblast growth factors (FGFs), vascular endothelial growth factors (VEGFs), and platelet-derived growth factors (PDGFs), coordinating the extracellular space distribution of these growth factors [119].

In order to understand the signaling properties and the array of functions of syndecan-4, it is necessary to look separately at the different domains of this molecule: the extracellular, the transmembrane, and the intracellular. The cleavage and shedding of the syndecan-4 extracellular domain, also termed ectodomain, play an important role in mediation of its extracellular signaling. The shed syndecan-4 ectodomain regulates cellular adhesion to the surrounding matrix and is capable of orchestrating the direct contact of cells with ECM proteins [34, 122]. Shedding of this domain leads to the dissipation of extracellular SOD, heparin-binding growth factors, and a host of other biologically active substances mentioned above, which are concentrated within EG. The loss of their surface gradient compromises cellular defense against oxidative stress, reduces growth and pro-survival signaling, and impairs interactions with other cells [34].

At the transmembrane site, syndecan-4 unfolds three signaling functions: it non-covalently clusters into sodium dodecyl sulfate (SDS)-resistant oligomers (the hallmark of lipid-rich domains, such as caveolae) which harbor diverse signaling cascades; it balances the interaction between growth factors, their cognate receptors, and other cell membrane receptors; and it serves as a direct link between the ECM and intracellular signaling proteins [34].

Intracellular domain of syndecan-4 and one of its major binding partners, synectin, facilitates the binding of Rho guanidine dissociation inhibitor 1 (RhoGDI1) and serves to insulate and decrease the activity of Rho family GTPases that are incorporated into the syndecan-4–synectin–RhoGDI1 complex at the cell membrane [33] in the absence of growth factor stimulation. Upon stimulation by growth factors, syndecan-4 reverses the described decrease in the activity of Rho family GTPases through its ability to bind and activate PKCα. PKCα in turn phosphorylates RhoGDI1 at Ser96, which allows the release of sequestered RhoG and Rac1 [34].

The role of proteoglycans and one of their major members, syndecan-4, in a variety of pathologic processes, has been a subject of a score of investigations. After skin injury, the expression of syndecan-4, on the one hand, is temporarily decreased in those keratinocytes, which migrate into the wound, and, on the other hand, it is increased in those keratinocytes, which proliferate at the wound margins, with specific increase in fibroblasts within the forming granulation tissue [32, 41]. Mice with a disrupted syndecan-4 gene have delayed healing of skin wounds and impaired angiogenesis in the granulation tissue [28]. Furthermore, it has been shown that syndecan-4^−/−^ mice have an increased mortality rate after a myocardial infarction due to cardiac rupture. Those cardiac events are associated with reduced inflammatory reaction and impaired granulation tissue formation due to reduced numbers of infiltrating leukocytes, fibroblasts, myofibroblasts, macrophages, and capillary vessels [80]. In addition, mice deficient in syndecan-4 have an increased susceptibility to LPS-injections manifested in increased mortality [55]. On this background, it has recently been reported that syndecan-4 knockout in mice protects against tubulointerstitial fibrosis apparently due to the reduction of tissue transglutaminase activity [106]. This finding has been substantiated by the study of Wee et al. showing the activation of transglutaminase 2 and syndecan-4 by tissue-resident natural killer cells in a model of aristolochic acid-induced nephropathy [129]. Contrasting with a wealth of the abovementioned critical functions of syndecan-4, the question of the mechanism responsible for its reported pro-fibrotic action in renal injury requires elucidation.

It has been argued that these harmful effects could be due, at least in part, to the effects of its extracellular ectodomain [115], rather than the effects of the whole syndecan-4 molecule. In fact, the mouse knockout model of syndecan-4 deficiency has been generated by deleting the exon encoding the extracellular domain [106]. The physiology of the syndecan-4 ectodomain is noteworthy because it is highly distinct from functions of the whole molecule, as mentioned previously. The ectodomain of syndecan-4 promotes collagen cross-linking and induces innate immunity signaling, stimulates immune cell infiltration, therefore exhibiting a central role in chemotaxis [114]. The release of the ectodomain is induced by pro-inflammatory stimuli, like, for instance, in the event of tissue injury [74, 114]. On the other hand, maintenance mechanisms exist to prevent the excessive release of the ectodomain. One important maintaining factor is endothelial SIRT1.

SIRT1 exhibits an important functional role in maintaining homeostasis of endothelial cells, and in reverse, SIRT1 deficiency in endothelial cells leads to a long list of endothelial abnormalities, as mentioned above. One of these abnormalities is the loss of EG. The expression of the EG is dramatically reduced in SIRT1^endo^-/- mice, a model of global endothelial dysfunction [73, 123]. In addition, syndecan-4 ectodomain is highly upregulated in this model of global endothelial dysfunction. In other words, the impairment of EG goes hand in hand with an impaired whole syndecan-4 molecule, leading to an increased amount of shed syndecan-4, allowing the syndecan-4 ectodomain to be released and to participate in fibrogenesis. How does this occur?

**Fig. 2:**
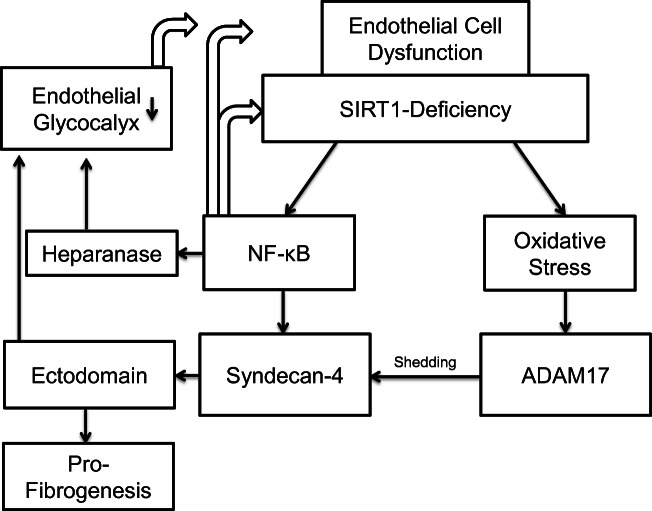
Endothelial Sirtuin1-deficiency leads to the degradation of the endothelial glycocalyx. Endothelial Sirtuin1-deficiency, a model of global endothelial dysfunction, leads on the one hand to increase NF-κB signaling and on the other hand causes increased oxidative stress. The increased NF-κB signaling induces both the transcription of syndecan-4 and heparanase. In addition to that NF-κB reduces Sirtuin1-activity and therefore sustaining the endothelial cell dysfunction. The increased oxidative stress induces ADAM17-acitivity which results in a greater amount of shedding of syndecan-4. The higher shedding of syndecan-4 and the higher activity of heparanase cause the degradation of the endothelial glycocalyx. Furthermore, the shed ectodomain of syndecan-4 gains systemic effects and potentiates a pro-fibrogenic response

Interestingly, syndecan-4 is a NF-κB target gene [115]. As described above, SIRT-1 deacetylates p65 protein preventing the nucleus translocation of NF-κB [12, 131]. Hence, increased SIRT-1 activity results in the inhibition of NF-κB-dependent inflammatory reactions [93, 131] while the decreased SIRT-1 activity enhances NF-κB signaling [58]. Indeed, our studies showed that the nuclear translocation of NF-kB is increased and syndecan-4 transcripts are elevated in SIRT1^endo^-/- mice, whereas the expression of syndecan-4 on the surface of renal microvascular endothelial cells isolated from SIRT1^endo^-/- mice was found to be decreased in parallel with increased syndecan-4 ectodomain abundance in the secretome of these cells and in the interstitium of the kidneys undergoing fibrotic transformation after an experimental insult [73]. Apparently, despite the elevated message level, syndecan-4 ectodomain is shed from the endothelial surface of SIRT1^endo^-/- mice, thus depleting the major scaffolding component of EG. These relations between SIRT-1 and NF-κB may explain the degradation of the EG in SIRT1^endo^-/- mice, resulting in a higher amount of shedding of syndecan-4 (Fig. 2).

In addition to that, we have observed that renal microvascular endothelial cells isolated from SIRT1^endo^-/- mice have an increased basal and stimulated superoxide generation. This finding is consistent with the known effect of SIRT1 to deacetylate Forkhead box O (FoxO1) DNA-binding proteins. The deacetylated form is necessary for the post-translational modification of this transcription factor which is needed for its active modification and higher cellular defense against oxidative stress [15, 83]. In turn, increased oxidative stress in renal microvascular endothelial cells of SIRT1^endo^-/- mice further leads to the activation of a redox-sensitive domain of the sheddase cleaving the ectodomain of syndecan-4, disintegrin, and metalloproteinase domain-containing protein 17 (ADAM-17) [128]. Activation of ADAM-17 may be ultimately responsible for shedding of syndecan-4 [60].

## Sheddases targeting the endothelial glycocalyx

Increased shedding of the EG components has been linked to the pathogenesis of a wide variety of diseases. There is data demonstrating the degradation of the EG in the course of hypertensive diseases or in the hemolytic uremic syndrome, as judged by the detection of increased amounts of shed heparan sulfates, syndecans, and hyaluronan into the bloodstream or the urine [8]. In a cohort study, it has been shown that trauma patients demonstrated a higher amount of shed EG components (syndecan-1, hyaluronic acid). The higher amount of shedding has been linked to a lower plasma colloid osmotic pressure, indicating a correlation between low plasma colloid osmotic pressure and degradation of the EG [100]. A different study with a similar approach disclosed the correlation between an increased release of atrial natriuretic peptide and higher plasma levels of EG components (hyaluronan, heparan sulfate, and syndecan-1) during hypervolemia [18]. There is also a hypothesis that the degradation of the EG manifests as an important step in the pathogenesis of malaria. Hempel et al. proposed that infected erythrocytes bind to the outer layer of the EG leading to increased shedding and allowing the parasites to interact with proteins in the deeper layer of the EG [51].

Shedding of the EG also causes biologically active components and proteins bound to the endothelial surface layer to disappear from the close vicinity of the luminal vascular surface, with the consequent loss of their respective local actions and gain of systemic cytokine-like effects [6]. For example, the loss of xanthine oxidoreductase at the endothelial surface leads to decreased production of uric acid [4], less lipoprotein lipase limits the activity of the lipid metabolism which further limits the number of chylomicrons, and free fatty acids to parenchymal cells [62, 105]. Shedding of syndecan-4, as described in detail previously, provides a good example for the acquisition by the ectodomain of systemic effects.

Investigations of the shedding of the EG have been the target of a score of studies, and the results of these studies suggest that matrix metalloproteinases (MMPs) are the major contributors to cleaving scaffolding molecular components of the EG and, therefore, facilitating shedding under pathological circumstances [69]. MMPs are calcium-dependent zinc-containing endopeptidases that play an important role in tissue remodeling associated with morphogenesis, angiogenesis, wound healing, arthritis, and cancer [112, 124]. Once activated, MMPs degrade extracellular matrices (collagen, elastin, gelatin), induce cell migration by providing directional cues, create substrate-cleavage fragments, coordinate tissue architecture, and modify the activity of signaling molecules [113]. Specifically, MMP-2, MMP-7, and MMP-9 are capable of directly cleaving chondroitin sulfate [49] and MMP-1 cleaves the heparan sulfate proteoglycan syndecan-1 [35]. MMP-9 is also the major sheddase of syndecan-4 in glomerular endothelial cells in the setting of diabetic nephropathy [102]. Most importantly, both the active and proactive forms of MMP-2 and MMP-9 are stored in the vesicular compartment within endothelial cells, suggesting the existence of mechanisms by which MMPs can be rapidly released by these cells [117].

An additional important contributor to shedding of the EG is heparanase. Heparanase cleaves the glycosidic bond within the heparan sulfate chain at specific sites. It is synthesized as a pre-proheparanase, processed to proheparanase at the endoplasmic reticulum, and transported to the Golgi apparatus, where it is packaged into vesicles and finally secreted [46]. After secretion, heparanase interacts with cell membrane heparan sulfate proteoglycans (especially with syndecans), low density lipoprotein receptor-related proteins, and mannose 6-phosphate receptors [44]. Heparanase is involved in pathologic processes in tumor growth, angiogenesis, metastasis, inflammation, and glomerular diseases [88]. During the investigation of inflammatory settings, like vascular damage or rheumatoid arthritis, it has been discovered that heparanase expression occurs mainly in the epithelial and/or endothelial compartment [3, 46, 68] and is induced by inflammatory cytokines [21, 29, 61, 66, 107]. Its main mechansims during inflammation is a result of neutrophil recruitment and the modulation of proinflammatory macrophage action. The induction of heparanase by inflammatory cytokines leads to the loss of the EG and therefore to endothelial hyperpermeability resulting in a higher amount of extravasation of neutrophils [104, 107]. However, there are also reports of high levels of heparanase resulting in a decreased amount of extravasation of neutrophils [67, 104]. Increased levels of heparanase also result in a decreased level of cell-surface heparan sulfate, making the toll-like receptor more accessible and therefore increasing the activation of macrophages [16, 66]. The enzymatic activity of heparanase is well studied in multiple myeloma. Patients suffering from multiple myeloma were found to have an increased level of heparanase in the bone marrow plasma [61, 86], and over 90% of multiple myeloma patients had increased heparanase expression in gene array analysis [75].

Notably, during the progression of chronic kidney disease, heparanase exerts an important contributing role through its participation in renal fibrogenesis via controlling the epithelial-mesenchymal transition in renal tubular cells [79]. A recent study performed by Masola et al. showed protection against chronic kidney dysfunction by inhibiting heparanase [77]. Abassi et al. showed in their experiments more profound renal injuries in the ischemic reperfusion model in heparanase-overexpressing (Hpa-tg) mice [1]. They also demonstrated a higher number of biomarkers of epithelial mesenchymal transition in Hpa-tg mice, suggesting heparanase to be of importance in the process of kidney fibrosis. An assumption which the same group corroborated in a study shows high levels of epithelial mesenchymal transition markers in wild-type mice, but no significant increase in those markers in heparanase-silenced tubular cells of the kidney [78]. As heparanase is also involved in the development of acute experimental glomerulonephritis via reinforcing renal leukocyte and macrophage influx by shrinking the heparan sulfate expression at the glomerulus [42], it is a great target for further clinical investigation.

Another type of sheddase is hyaluronidase. Hyaluronidase cleaves hyaluronan, a high-molecular weight, unsulfated glycosaminoglycan which is highly enriched on the apical surface of endothelial cells [30, 52]. It has been shown that there is an association between an increased hyaluronan metabolism and structural changes of the arterial wall with accelerated atherogenesis in type 1 diabetes as a result of heightened activity of hyaluronidase [89]. Furthermore, hyaluronidase has been implicated as a marker for various cancers, such as genitourinary, colorectal, or breast cancer [31, 65, 81, 96]. Because of being an inducer of pro-tumorigenic and pro-angiogenic phenotypes, therapeutic approaches targeting hyaluronidase are under intense investigation. So far partially sulfated hyaluronic acid polymers have been the only hyaluronidase inhibitors to be tested in an in vivo model. Benitez et al. showed delayed tumor growth in mice suffering of prostate cancer [7]. HA shed by hyaluronidase and measured in the plasma or urine is considered to be a marker of the integrity of the EG during sepsis, ischemia/reperfusion, and diabetes [26].

Important participants in the process of shedding of the EG are members of the ADAM family. The ADAM gene family is a member of a metalloproteinase superfamily, which includes a diverse group of multi-domain transmembrane and secreted proteins with a variety of biological functions. The main function of ADAM proteins is to regulate shedding of the extracellular domains of several proteins, such as tumor necrosis factor-α (TNFα) receptor, Notch, or transforming growth factor-α (TGFα) [14]. Specifically, ADAM17 has been shown to be involved in the degradation of the EG by shedding the ectodomain of syndecan-4 [94]. ADAM17 consists of three different domains: the prodomain, the catalytic domain, and the cytoplasmic domain. The catalytic domain is responsible for shedding and therefore it is the target structure for inhibition of ADAM17 [48]. ADAM17 has been implicated in a variety of diseases. As a TNFα-receptor-cleaving enzyme, ADAM17 has been shown to be upregulated in inflammatory diseases like rheumatoid arthritis or psoriasis [59, 92]. Because of its ability to shed growth factors, which are necessary for tumor growth and progression, ADAM17 has been reported to contribute to the development of a malignant phenotype [48]. Indeed, ADAM17 has been found to be overexpressed in breast and ovarian tumors [11, 116]. Involvement of ADAM17 in the setting of diabetes is most likely due to TNFα-activation through ADAM17 since treatment with an ADAM17 inhibitor improves insulin sensitivity [120]. In the context of the present discussion, ADAM17 activity is redox-dependent and oxidative stress enhances it [128]. Notably, renal microvascular endothelial cells isolated from SIRT1^endo^-/- mice exhibit a significant increase in basal and inducible generation of superoxide [73], thus potentially explaining the elevated activity of ADAM17 in these cells.

Having listed the sheddases with the known specific targeting of the EG, the question arises if inhibition of these enzymes could preserve the integrity of the EG. For instance, experimental studies revealed protection by antithrombin against shedding of the EG under ischaemic and inflammatory conditions [19]. The antithrombin molecule consists of a binding domain for heparin and heparan, which is responsible for its anti-inflammatory effects, besides inhibiting serine proteases [127]. In other words, antithrombin prevents degradation of the endothelial glycocalyx, because of a tight binding to heparan sulfate proteoglycans and, therefore, blocking access of sheddases.

A different approach would be to inhibit the above mentioned sheddases directly. In a clinical study, inhibition of ADAM17 reduced the presence of the epidermal growth factor receptor ligand TGF-α, which is incriminated in renal fibrosis. Therefore, inhibition of ADAM17 has the potential to intervene in human renal diseases [82]. Furthermore, Hu et al. showed that intravenous injection of a MMP-9 inhibitor protects mice against lethal endotoxin shock [54]. Zeng et al. demonstrated protection of the EG through sphingosine-1-phosphate mediated inhibition of syndecan-1 shedding [132].

## Summary

In summary, endothelial SIRT1-deficiency, a model of global endothelial dysfunction, leads to upregulation of syndecan-4 and release of its ectodomain, the loss of the EG through increased superoxide generation and induced NF-κB signaling. Furthermore syndecan-4, a major proteoglycan component of the EG and a binding partner of glycosaminoglycans comprising this structure are shed to a greater extent and its released ectodomain acts as a pro-fibrotic molecule, coordinating the inflammatory and pro-fibrogenic responses after applied damage to the endothelium and the consequent loss of the EG. The data and arguments presented herein provide a tentative outline of a potentially new target of SIRT1, endothelial glycocalyx, and emphasize the role of defective SIRT1 deacetylation in glycocalyx degradation.

## References

[1] Abassi Z, Hamoud S, Hassan A, Khamaysi I, Nativ O, Heyman SN, Muhammad RS, Ilan N, Singh P, Hammond E, Zaza G, Lupo A, Onisto M, Bellin G, Masola V, Vlodavsky I, Gambaro G (2017). Involvement of heparanase in the pathogenesis of acute kidney injury: nephroprotective effect of PG545. Oncotarget.

[2] Annecke T, Fischer J, Hartmann H, Tschoep J, Rehm M, Conzen P, Sommerhoff CP, Becker BF (2011). Shedding of the coronary endothelial glycocalyx: effects of hypoxia/reoxygenation vs ischaemia/reperfusion. Br J Anaesth.

[3] Baker AB, Groothuis A, Jonas M, Ettenson DS, Shazly T, Zcharia E, Vlodavsky I, Seifert P, Edelman ER (2009). Heparanase alters arterial structure, mechanics, and repair following endovascular stenting in mice. Circ Res.

[4] Becker BF (1993). Towards the physiological function of uric acid. Free Radic Biol Med.

[5] Becker BF, Chappell D, Jacob M (2010). Endothelial glycocalyx and coronary vascular permeability: the fringe benefit. Basic Res Cardiol.

[6] Becker BF, Jacob M, Leipert S, Salmon AHJ, Chappell D (2015). Degradation of the endothelial glycocalyx in clinical settings: searching for the sheddases. Br J Clin Pharmacol.

[7] Benitez A, Yates TJ, Lopez LE, Cerwinka WH, Bakkar A, Lokeshwar VB (2011). Targeting hyaluronidase for cancer therapy: antitumor activity of sulfated hyaluronic acid in prostate cancer cells. Cancer Res.

[8] Boels MGS, Lee DH, van den Berg BM, Dane MJC, van der Vlag J, Rabelink TJ (2013). The endothelial glycocalyx as a potential modifier of the hemolytic uremic syndrome. Eur J Intern Med.

[9] Bombeli T, Schwartz BR, Harlan JM (1998). Adhesion of activated platelets to endothelial cells: evidence for a GPIIbIIIa-dependent bridging mechanism and novel roles for endothelial intercellular adhesion molecule 1 (ICAM-1), alphavbeta3 integrin, and GPIbalpha. J Exp Med.

[10] Bonizzi G, Karin M (2004). The two NF-kappaB activation pathways and their role in innate and adaptive immunity. Trends Immunol.

[11] Borrell-Pagès M, Rojo F, Albanell J, Baselga J, Arribas J (2003). TACE is required for the activation of the EGFR by TGF-alpha in tumors. EMBO J.

[12] Bourguignon LYW, Xia W, Wong G (2009). Hyaluronan-mediated CD44 interaction with p300 and SIRT1 regulates beta-catenin signaling and NFkappaB-specific transcription activity leading to MDR1 and Bcl-xL gene expression and chemoresistance in breast tumor cells. J Biol Chem.

[13] Brasier AR (2006). The NF-kappaB regulatory network. Cardiovasc Toxicol.

[14] Brocker CN, Vasiliou V, Nebert DW (2009). Evolutionary divergence and functions of the ADAM and ADAMTS gene families. Hum Genomics.

[15] Brunet A, Sweeney LB, Sturgill JF, Chua KF, Greer PL, Lin Y, Tran H, Ross SE, Mostoslavsky R, Cohen HY, Hu LS, Cheng H-L, Jedrychowski MP, Gygi SP, Sinclair DA, Alt FW, Greenberg ME (2004). Stress-dependent regulation of FOXO transcription factors by the SIRT1 deacetylase. Science.

[16] Brunn GJ, Bungum MK, Johnson GB, Platt JL (2005). Conditional signaling by toll-like receptor 4. FASEB J.

[17] Chalkiadaki A, Guarente L (2015). The multifaceted functions of sirtuins in cancer. Nat Rev Cancer.

[18] Chappell D, Bruegger D, Potzel J, Jacob M, Brettner F, Vogeser M, Conzen P, Becker BF, Rehm M (2014). Hypervolemia increases release of atrial natriuretic peptide and shedding of the endothelial glycocalyx. Crit Care.

[19] Chappell D, Jacob M, Hofmann-Kiefer K, Rehm M, Welsch U, Conzen P, Becker BF (2009). Antithrombin reduces shedding of the endothelial glycocalyx following ischaemia/reperfusion. Cardiovasc Res.

[20] Chen FE, Huang DB, Chen YQ, Ghosh G (1998). Crystal structure of p50/p65 heterodimer of transcription factor NF-kappaB bound to DNA. Nature.

[21] Chen G, Wang D, Vikramadithyan R, Yagyu H, Saxena U, Pillarisetti S, Goldberg IJ (2004). Inflammatory cytokines and fatty acids regulate endothelial cell heparanase expression. Biochemistry.

[22] Chen J, Xavier S, Moskowitz-Kassai E, Chen R, Lu CY, Sanduski K, Špes A, Turk B, Goligorsky MS (2012). Cathepsin cleavage of sirtuin 1 in endothelial progenitor cells mediates stress-induced premature senescence. Am J Pathol.

[23] Couchman JR (2003). Syndecans: proteoglycan regulators of cell-surface microdomains?. Nat Rev Mol Cell Biol.

[24] Couchman JR (2010). Transmembrane signaling proteoglycans. Annu Rev Cell Dev Biol.

[25] Deckert T, Horowitz IM, Kofoed-Enevoldsen A, Kjellén L, Deckert M, Lykkelund C, Burcharth F (1991). Possible genetic defects in regulation of glycosaminoglycans in patients with diabetic nephropathy. Diabetes.

[26] Dogné S, Flamion B (2020). Endothelial glycocalyx impairment in disease: focus on hyaluronan shedding. Am J Pathol.

[27] Dragovich MA, Chester D, Fu BM, Wu C, Xu Y, Goligorsky MS, Zhang XF (2016). Mechanotransduction of the endothelial glycocalyx mediates nitric oxide production through activation of TRP channels. Am J Physiol, Cell Physiol.

[28] Echtermeyer F, Streit M, Wilcox-Adelman S, Saoncella S, Denhez F, Detmar M, Goetinck P (2001). Delayed wound repair and impaired angiogenesis in mice lacking syndecan-4. J Clin Invest.

[29] Edovitsky E, Lerner I, Zcharia E, Peretz T, Vlodavsky I, Elkin M (2006). Role of endothelial heparanase in delayed-type hypersensitivity. Blood.

[30] Eggli PS, Graber W (1995). Association of hyaluronan with rat vascular endothelial and smooth muscle cells. J Histochem Cytochem.

[31] Eissa S, Swellam M, Shehata H, El-Khouly IM, El-Zayat T, El-Ahmady O (2010). Expression of HYAL1 and survivin RNA as diagnostic molecular markers for bladder cancer. J Urol.

[32] Elenius K, Vainio S, Laato M, Salmivirta M, Thesleff I, Jalkanen M (1991). Induced expression of syndecan in healing wounds. J Cell Biol.

[33] Elfenbein A, Rhodes JM, Meller J, Schwartz MA, Matsuda M, Simons M (2009). Suppression of RhoG activity is mediated by a syndecan 4-synectin-RhoGDI1 complex and is reversed by PKCalpha in a Rac1 activation pathway. J Cell Biol.

[34] Elfenbein A, Simons M (2013). Syndecan-4 signaling at a glance. J Cell Sci.

[35] Endo K, Takino T, Miyamori H, Kinsen H, Yoshizaki T, Furukawa M, Sato H (2003). Cleavage of syndecan-1 by membrane type matrix metalloproteinase-1 stimulates cell migration. J Biol Chem.

[36] Eriksson AS, Spillmann D (2012). The mutual impact of syndecan-1 and its glycosaminoglycan chains--a multivariable puzzle. J Histochem Cytochem.

[37] Fan J, Sun Y, Xia Y, Tarbell JM, Fu BM (2019). Endothelial surface glycocalyx (ESG) components and ultra-structure revealed by stochastic optical reconstruction microscopy (STORM). Biorheology.

[38] Florian JA, Kosky JR, Ainslie K, Pang Z, Dull RO, Tarbell JM (2003). Heparan sulfate proteoglycan is a mechanosensor on endothelial cells. Circ Res.

[39] Frye RA (1999). Characterization of five human cDNAs with homology to the yeast SIR2 gene: Sir2-like proteins (sirtuins) metabolize NAD and may have protein ADP-ribosyltransferase activity. Biochem Biophys Res Commun.

[40] Fu BM, Tarbell JM (2013). Mechano-sensing and transduction by endothelial surface glycocalyx: composition, structure, and function. Wiley Interdiscip Rev Syst Biol Med.

[41] Gallo R, Kim C, Kokenyesi R, Adzick NS, Bernfield M (1996). Syndecans-1 and -4 are induced during wound repair of neonatal but not fetal skin. J Invest Dermatol.

[42] Garsen M, Benner M, Dijkman HB, van Kuppevelt TH, Li J-P, Rabelink TJ, Vlodavsky I, Berden JHM, Rops ALWMM, Elkin M, van der Vlag J (2016). Heparanase is essential for the development of acute experimental glomerulonephritis. Am J Pathol.

[43] Garsen M, Lenoir O, Rops ALWMM, Dijkman HB, Willemsen B, van Kuppevelt TH, Rabelink TJ, Berden JHM, Tharaux P-L, van der Vlag J (2016). Endothelin-1 induces proteinuria by heparanase-mediated disruption of the glomerular glycocalyx. J Am Soc Nephrol.

[44] Garsen M, Rops ALWMM, Rabelink TJ, Berden JHM, van der Vlag J (2014). The role of heparanase and the endothelial glycocalyx in the development of proteinuria. Nephrol Dial Transplant.

[45] Ghosh S, May MJ, Kopp EB (1998). NF-kappa B and Rel proteins: evolutionarily conserved mediators of immune responses. Annu Rev Immunol.

[46] Goldberg R, Meirovitz A, Hirshoren N, Bulvik R, Binder A, Rubinstein AM, Elkin M (2013). Versatile role of heparanase in inflammation. Matrix Biol.

[47] Goligorsky MS, Sun D (2020). Glycocalyx in Endotoxemia and Sepsis. Am J Pathol.

[48] Gooz M (2010). ADAM-17: the enzyme that does it all. Crit Rev Biochem Mol Biol.

[49] Gronski TJ, Martin RL, Kobayashi DK, Walsh BC, Holman MC, Huber M, Van Wart HE, Shapiro SD (1997). Hydrolysis of a broad spectrum of extracellular matrix proteins by human macrophage elastase. J Biol Chem.

[50] Guarente L (2011). Franklin H. Epstein lecture: sirtuins, aging, and medicine. N Engl J Med.

[51] Hempel C, Pasini EM, Kurtzhals JAL (2016). Endothelial glycocalyx: shedding light on malaria pathogenesis. Trends Mol Med.

[52] Henry CB, Duling BR (1999). Permeation of the luminal capillary glycocalyx is determined by hyaluronan. Am J Phys.

[53] Houtkooper RH, Pirinen E, Auwerx J (2012). Sirtuins as regulators of metabolism and healthspan. Nat Rev Mol Cell Biol.

[54] Hu J, Van den Steen PE, Dillen C, Opdenakker G (2005). Targeting neutrophil collagenase/matrix metalloproteinase-8 and gelatinase B/matrix metalloproteinase-9 with a peptidomimetic inhibitor protects against endotoxin shock. Biochem Pharmacol.

[55] Ishiguro K, Kadomatsu K, Kojima T, Muramatsu H, Iwase M, Yoshikai Y, Yanada M, Yamamoto K, Matsushita T, Nishimura M, Kusugami K, Saito H, Muramatsu T (2001). Syndecan-4 deficiency leads to high mortality of lipopolysaccharide-injected mice. J Biol Chem.

[56] Ju R, Zhuang ZW, Zhang J, Lanahan AA, Kyriakides T, Sessa WC, Simons M (2014). Angiopoietin-2 secretion by endothelial cell exosomes: regulation by the phosphatidylinositol 3-kinase (PI3K)/Akt/endothelial nitric oxide synthase (eNOS) and syndecan-4/syntenin pathways. J Biol Chem.

[57] Jung U, Ley K (1997). Regulation of E-selectin, P-selectin, and intercellular adhesion molecule 1 expression in mouse cremaster muscle vasculature. Microcirculation.

[58] Kauppinen A, Suuronen T, Ojala J, Kaarniranta K, Salminen A (2013). Antagonistic crosstalk between NF-κB and SIRT1 in the regulation of inflammation and metabolic disorders. Cell Signal.

[59] Kawaguchi M, Mitsuhashi Y, Kondo S (2005). Overexpression of tumour necrosis factor-alpha-converting enzyme in psoriasis. Br J Dermatol.

[60] Kawahara R, Lima RN, Domingues RR, Pauletti BA, Meirelles GV, Assis M, Figueira ACM, Paes Leme AF (2014). Deciphering the role of the ADAM17-dependent secretome in cell signaling. J Proteome Res.

[61] Kelly T, Miao H-Q, Yang Y, Navarro E, Kussie P, Huang Y, MacLeod V, Casciano J, Joseph L, Zhan F, Zangari M, Barlogie B, Shaughnessy J, Sanderson RD (2003). High heparanase activity in multiple myeloma is associated with elevated microvessel density. Cancer Res.

[62] Kersten S (2014). Physiological regulation of lipoprotein lipase. Biochim Biophys Acta.

[63] Kida Y, Goligorsky MS (2016). Sirtuins, cell senescence, and vascular aging. Can J Cardiol.

[64] Kim CW, Goldberger OA, Gallo RL, Bernfield M (1994). Members of the syndecan family of heparan sulfate proteoglycans are expressed in distinct cell-, tissue-, and development-specific patterns. Mol Biol Cell.

[65] Kolliopoulos C, Bounias D, Bouga H, Kyriakopoulou D, Stavropoulos M, Vynios DH (2013). Hyaluronidases and their inhibitors in the serum of colorectal carcinoma patients. J Pharm Biomed Anal.

[66] Lerner I, Hermano E, Zcharia E, Rodkin D, Bulvik R, Doviner V, Rubinstein AM, Ishai-Michaeli R, Atzmon R, Sherman Y, Meirovitz A, Peretz T, Vlodavsky I, Elkin M (2011). Heparanase powers a chronic inflammatory circuit that promotes colitis-associated tumorigenesis in mice. J Clin Invest.

[67] Li L, Wang B, Gao T, Zhang X, Hao J-X, Vlodavsky I, Wiesenfeld-Hallin Z, Xu X-J, Li J-P (2012). Heparanase overexpression reduces carrageenan-induced mechanical and cold hypersensitivity in mice. Neurosci Lett.

[68] Li RW, Freeman C, Yu D, Hindmarsh EJ, Tymms KE, Parish CR, Smith PN (2008). Dramatic regulation of heparanase activity and angiogenesis gene expression in synovium from patients with rheumatoid arthritis. Arthritis Rheum.

[69] Lipowsky HH (2011). Protease activity and the role of the endothelial glycocalyx in inflammation. Drug Discov Today Dis Models.

[70] Lipphardt M, Dihazi H, Jeon NL, Dadafarin S, Ratliff BB, Rowe DW, Müller GA, Goligorsky MS (2019). Dickkopf-3 in aberrant endothelial secretome triggers renal fibroblast activation and endothelial-mesenchymal transition. Nephrol Dial Transplant.

[71] Lipphardt M, Dihazi H, Müller GA, Goligorsky MS (2018). Fibrogenic Secretome of Sirtuin 1-deficient endothelial cells: Wnt, notch and glycocalyx rheostat. Front Physiol.

[72] Lipphardt M, Song JW, Matsumoto K, Dadafarin S, Dihazi H, Müller G, Goligorsky MS (2017). The third path of tubulointerstitial fibrosis: aberrant endothelial secretome. Kidney Int.

[73] Lipphardt M, Song JW, Ratliff BB, Dihazi H, Müller GA, Goligorsky MS (2018). Endothelial dysfunction is a superinducer of syndecan-4: fibrogenic role of its ectodomain. Am J Physiol Heart Circ Physiol.

[74] Lunde IG, Herum KM, Carlson CC, Christensen G (2016). Syndecans in heart fibrosis. Cell Tissue Res.

[75] Mahtouk K, Hose D, Raynaud P, Hundemer M, Jourdan M, Jourdan E, Pantesco V, Baudard M, De Vos J, Larroque M, Moehler T, Rossi J-F, Rème T, Goldschmidt H, Klein B (2007). Heparanase influences expression and shedding of syndecan-1, and its expression by the bone marrow environment is a bad prognostic factor in multiple myeloma. Blood.

[76] Maizel J, Xavier S, Chen J, Lin CHS, Vasko R, Goligorsky MS (2014). Sirtuin 1 ablation in endothelial cells is associated with impaired angiogenesis and diastolic dysfunction. Am J Physiol Heart Circ Physiol.

[77] Masola V, Bellin G, Vischini G, Dall’Olmo L, Granata S, Gambaro G, Lupo A, Onisto M, Zaza G (2018). Inhibition of heparanase protects against chronic kidney dysfunction following ischemia/reperfusion injury. Oncotarget.

[78] Masola V, Zaza G, Gambaro G, Onisto M, Bellin G, Vischini G, Khamaysi I, Hassan A, Hamoud S, Nativ O, Heyman S, Lupo A, Vlodavsky I, Abassi Z (2016). Heparanase: a potential new factor involved in the renal epithelial mesenchymal transition (EMT) induced by ischemia/reperfusion (I/R) injury. PLoS One.

[79] Masola V, Zaza G, Onisto M, Lupo A, Gambaro G (2015). Impact of heparanase on renal fibrosis. J Transl Med.

[80] Matsui Y, Ikesue M, Danzaki K, Morimoto J, Sato M, Tanaka S, Kojima T, Tsutsui H, Uede T (2011). Syndecan-4 prevents cardiac rupture and dysfunction after myocardial infarction. Circ Res.

[81] McAtee CO, Barycki JJ, Simpson MA (2014). Emerging roles for hyaluronidase in cancer metastasis and therapy. Adv Cancer Res.

[82] Melenhorst WB, Visser L, Timmer A, van den Heuvel MC, Stegeman CA, van Goor H (2009). ADAM17 upregulation in human renal disease: a role in modulating TGF-alpha availability?. Am J Physiol Renal Physiol.

[83] Motta MC, Divecha N, Lemieux M, Kamel C, Chen D, Gu W, Bultsma Y, McBurney M, Guarente L (2004). Mammalian SIRT1 represses forkhead transcription factors. Cell.

[84] Mulivor AW, Lipowsky HH (2004). Inflammation- and ischemia-induced shedding of venular glycocalyx. Am J Physiol Heart Circ Physiol.

[85] Myrup B, Hansen PM, Jensen T, Kofoed-Enevoldsen A, Feldt-Rasmussen B, Gram J, Kluft C, Jespersen J, Deckert T (1995). Effect of low-dose heparin on urinary albumin excretion in insulin-dependent diabetes mellitus. Lancet.

[86] Nadir Y, Brenner B (2014). Heparanase multiple effects in cancer. Thromb Res.

[87] Nandi A, Estess P, Siegelman MH (2000). Hyaluronan anchoring and regulation on the surface of vascular endothelial cells is mediated through the functionally active form of CD44. J Biol Chem.

[88] Nasser NJ (2008). Heparanase involvement in physiology and disease. Cell Mol Life Sci.

[89] Nieuwdorp M, Holleman F, de Groot E, Vink H, Gort J, Kontush A, Chapman MJ, Hutten BA, Brouwer CB, Hoekstra JBL, Kastelein JJP, Stroes ESG (2007). Perturbation of hyaluronan metabolism predisposes patients with type 1 diabetes mellitus to atherosclerosis. Diabetologia.

[90] Nieuwdorp M, Meuwese MC, Vink H, Hoekstra JBL, Kastelein JJP, Stroes ESG (2005). The endothelial glycocalyx: a potential barrier between health and vascular disease. Curr Opin Lipidol.

[91] Nieuwdorp M, Mooij HL, Kroon J, Atasever B, Spaan JAE, Ince C, Holleman F, Diamant M, Heine RJ, Hoekstra JBL, Kastelein JJP, Stroes ESG, Vink H (2006). Endothelial glycocalyx damage coincides with microalbuminuria in type 1 diabetes. Diabetes.

[92] Ohta S, Harigai M, Tanaka M, Kawaguchi Y, Sugiura T, Takagi K, Fukasawa C, Hara M, Kamatani N (2001). Tumor necrosis factor-alpha (TNF-alpha) converting enzyme contributes to production of TNF-alpha in synovial tissues from patients with rheumatoid arthritis. J Rheumatol.

[93] Pan W, Yu H, Huang S, Zhu P (2016). Resveratrol protects against TNF-α-induced injury in human umbilical endothelial cells through promoting Sirtuin-1-induced repression of NF-KB and p38 MAPK. PLoS One.

[94] Piperigkou Z, Mohr B, Karamanos N, Götte M (2016). Shed proteoglycans in tumor stroma. Cell Tissue Res.

[95] Platts SH, Linden J, Duling BR (2003). Rapid modification of the glycocalyx caused by ischemia-reperfusion is inhibited by adenosine A2A receptor activation. Am J Physiol Heart Circ Physiol.

[96] Poola I, Abraham J, Marshalleck JJ, Yue Q, Lokeshwar VB, Bonney G, Dewitty RL (2008). Molecular risk assessment for breast cancer development in patients with ductal hyperplasias. Clin Cancer Res.

[97] Potente M, Ghaeni L, Baldessari D, Mostoslavsky R, Rossig L, Dequiedt F, Haendeler J, Mione M, Dejana E, Alt FW, Zeiher AM, Dimmeler S (2007). SIRT1 controls endothelial angiogenic functions during vascular growth. Genes Dev.

[98] Potter DR, van Teeffelen J, Vink H, van den Berg BM (2015). Perturbed mechanotransduction by endothelial surface glycocalyx modification greatly impairs the arteriogenic process. Am J Physiol Heart Circ Physiol.

[99] Pries AR, Secomb TW, Gaehtgens P (2000). The endothelial surface layer. Pflugers Arch.

[100] Rahbar E, Cardenas JC, Baimukanova G, Usadi B, Bruhn R, Pati S, Ostrowski SR, Johansson PI, Holcomb JB, Wade CE (2015). Endothelial glycocalyx shedding and vascular permeability in severely injured trauma patients. J Transl Med.

[101] Ramnath R, Foster RR, Qiu Y, Cope G, Butler MJ, Salmon AH, Mathieson PW, Coward RJ, Welsh GI, Satchell SC (2014). Matrix metalloproteinase 9-mediated shedding of syndecan 4 in response to tumor necrosis factor α: a contributor to endothelial cell glycocalyx dysfunction. FASEB J.

[102] Reine TM, Lanzalaco F, Kristiansen O, Enget AR, Satchell S, Jenssen TG, Kolset SO (2019) Matrix metalloproteinase-9 mediated shedding of syndecan-4 in glomerular endothelial cells. Microcirculation e12534. 10.1111/micc.12534 10.1111/micc.12534 30703289

[103] Reitsma S, Slaaf DW, Vink H, van Zandvoort MAMJ, oude MGA E (2007). The endothelial glycocalyx: composition, functions, and visualization. Pflugers Arch.

[104] Sanderson RD, Elkin M, Rapraeger AC, Ilan N, Vlodavsky I (2017). Heparanase regulation of cancer, autophagy and inflammation: new mechanisms and targets for therapy. FEBS J.

[105] Sarrazin S, Lamanna WC, Esko JD (2011) Heparan sulfate proteoglycans. Cold Spring Harb Perspect Biol 3. 10.1101/cshperspect.a004952 10.1101/cshperspect.a004952 PMC3119907 21690215

[106] Scarpellini A, Huang L, Burhan I, Schroeder N, Funck M, Johnson TS, Verderio EAM (2014). Syndecan-4 knockout leads to reduced extracellular transglutaminase-2 and protects against tubulointerstitial fibrosis. J Am Soc Nephrol.

[107] Schmidt EP, Yang Y, Janssen WJ, Gandjeva A, Perez MJ, Barthel L, Zemans RL, Bowman JC, Koyanagi DE, Yunt ZX, Smith LP, Cheng SS, Overdier KH, Thompson KR, Geraci MW, Douglas IS, Pearse DB, Tuder RM (2012). The pulmonary endothelial glycocalyx regulates neutrophil adhesion and lung injury during experimental sepsis. Nat Med.

[108] Snoeijs MG, Vink H, Voesten N, Christiaans MH, Daemen J-WH, Peppelenbosch AG, Tordoir JH, Peutz-Kootstra CJ, Buurman WA, Schurink GWH, van Heurn LWE (2010). Acute ischemic injury to the renal microvasculature in human kidney transplantation. Am J Physiol Renal Physiol.

[109] Song JW, Zullo J, Lipphardt M, Dragovich M, Zhang FX, Fu B, Goligorsky MS (2018). Endothelial glycocalyx-the battleground for complications of sepsis and kidney injury. Nephrol Dial Transplant.

[110] Song JW, Zullo JA, Liveris D, Dragovich M, Zhang XF, Goligorsky MS (2017). Therapeutic restoration of endothelial glycocalyx in sepsis. J Pharmacol Exp Ther.

[111] Sperandio M (2006). Selectins and glycosyltransferases in leukocyte rolling in vivo. FEBS J.

[112] Spinale FG (2007). Myocardial matrix remodeling and the matrix metalloproteinases: influence on cardiac form and function. Physiol Rev.

[113] Sternlicht MD, Werb Z (2001). How matrix metalloproteinases regulate cell behavior. Annu Rev Cell Dev Biol.

[114] Strand ME, Aronsen JM, Braathen B, Sjaastad I, Kvaløy H, Tønnessen T, Christensen G, Lunde IG (2015). Shedding of syndecan-4 promotes immune cell recruitment and mitigates cardiac dysfunction after lipopolysaccharide challenge in mice. J Mol Cell Cardiol.

[115] Strand ME, Herum KM, Rana ZA, Skrbic B, Askevold ET, Dahl CP, Vistnes M, Hasic A, Kvaløy H, Sjaastad I, Carlson CR, Tønnessen T, Gullestad L, Christensen G, Lunde IG (2013). Innate immune signaling induces expression and shedding of the heparan sulfate proteoglycan syndecan-4 in cardiac fibroblasts and myocytes, affecting inflammation in the pressure-overloaded heart. FEBS J.

[116] Tanaka Y, Miyamoto S, Suzuki SO, Oki E, Yagi H, Sonoda K, Yamazaki A, Mizushima H, Maehara Y, Mekada E, Nakano H (2005). Clinical significance of heparin-binding epidermal growth factor-like growth factor and a disintegrin and metalloprotease 17 expression in human ovarian cancer. Clin Cancer Res.

[117] Taraboletti G, D’Ascenzo S, Borsotti P, Giavazzi R, Pavan A, Dolo V (2002). Shedding of the matrix metalloproteinases MMP-2, MMP-9, and MT1-MMP as membrane vesicle-associated components by endothelial cells. Am J Pathol.

[118] Teng YH-F, Aquino RS, Park PW (2012). Molecular functions of syndecan-1 in disease. Matrix Biol.

[119] Tkachenko E, Rhodes JM, Simons M (2005). Syndecans: new kids on the signaling block. Circ Res.

[120] Togashi N, Ura N, Higashiura K, Murakami H, Shimamoto K (2002). Effect of TNF-alpha--converting enzyme inhibitor on insulin resistance in fructose-fed rats. Hypertension.

[121] Tornatore L, Thotakura AK, Bennett J, Moretti M, Franzoso G (2012). The nuclear factor kappa B signaling pathway: integrating metabolism with inflammation. Trends Cell Biol.

[122] Tumova S, Woods A, Couchman JR (2000). Heparan sulfate chains from glypican and syndecans bind the Hep II domain of fibronectin similarly despite minor structural differences. J Biol Chem.

[123] Vasko R, Xavier S, Chen J, Lin CHS, Ratliff B, Rabadi M, Maizel J, Tanokuchi R, Zhang F, Cao J, Goligorsky MS (2014). Endothelial sirtuin 1 deficiency perpetrates nephrosclerosis through downregulation of matrix metalloproteinase-14: relevance to fibrosis of vascular senescence. J Am Soc Nephrol.

[124] Verma RP, Hansch C (2007). Matrix metalloproteinases (MMPs): chemical-biological functions and (Q)SARs. Bioorg Med Chem.

[125] Vuong TT, Reine TM, Sudworth A, Jenssen TG, Kolset SO (2015). Syndecan-4 is a major syndecan in primary human endothelial cells in vitro, modulated by inflammatory stimuli and involved in wound healing. J Histochem Cytochem.

[126] Wang G, Tiemeier GL, van den Berg BM, Rabelink TJ (2020). Endothelial glycocalyx hyaluronan: regulation and role in prevention of diabetic complications. Am J Pathol.

[127] Wang J, Wang Y, Wang J, Gao J, Tong C, Manithody C, Li J, Rezaie AR (2013). Antithrombin is protective against myocardial ischemia and reperfusion injury. J Thromb Haemost.

[128] Wang Y, Herrera AH, Li Y, Belani KK, Walcheck B (2009). Regulation of mature ADAM17 by redox agents for L-selectin shedding. J Immunol.

[129] Wee YM, Go H, Choi MY, Jung HR, Cho YM, Kim YH, Han DJ, Shin S (2019). Tissue-resident natural killer cells exacerbate tubulointerstitial fibrosis by activating transglutaminase 2 and syndecan-4 in a model of aristolochic acid-induced nephropathy. BMB Rep.

[130] Xie J, Zhang X, Zhang L (2013). Negative regulation of inflammation by SIRT1. Pharmacol Res.

[131] Yeung F, Hoberg JE, Ramsey CS, Keller MD, Jones DR, Frye RA, Mayo MW (2004). Modulation of NF-kappaB-dependent transcription and cell survival by the SIRT1 deacetylase. EMBO J.

[132] Zeng Y, Adamson RH, Curry F-RE, Tarbell JM (2014). Sphingosine-1-phosphate protects endothelial glycocalyx by inhibiting syndecan-1 shedding. Am J Physiol Heart Circ Physiol.

[133] Zhang X, Sun D, Song JW, Zullo J, Lipphardt M, Coneh-Gould L, Goligorsky MS (2018). Endothelial cell dysfunction and glycocalyx - a vicious circle. Matrix Biol.

[134] Zullo JA, Fan J, Azar TT, Yen W, Zeng M, Chen J, Ratliff BB, Song J, Tarbell JM, Goligorsky MS, Fu BM (2016). Exocytosis of endothelial lysosome-related organelles hair-triggers a patchy loss of glycocalyx at the onset of sepsis. Am J Pathol.

